# MicroRNAs as Factors in Bidirectional Crosstalk Between Mitochondria and the Nucleus During Cellular Senescence

**DOI:** 10.3389/fphys.2021.734976

**Published:** 2021-09-09

**Authors:** Chiara Giordani, Andrea Silvestrini, Angelica Giuliani, Fabiola Olivieri, Maria Rita Rippo

**Affiliations:** ^1^Department of Clinical and Molecular Sciences, DISCLIMO, Università Politecnica delle Marche, Ancona, Italy; ^2^Center of Clinical Pathology and Innovative Therapy, IRCCS INRCA, Ancona, Italy

**Keywords:** microRNA, senescence, mitochondria, mitonuclear communication, mitomiRs

## Abstract

Mitochondria are essential organelles that generate most of the chemical energy to power the cell through ATP production, thus regulating cell homeostasis. Although mitochondria have their own independent genome, most of the mitochondrial proteins are encoded by nuclear genes. An extensive bidirectional communication network between mitochondria and the nucleus has been discovered, thus making them semi-autonomous organelles. The nucleus-to-mitochondria signaling pathway, called Anterograde Signaling Pathway can be deduced, since the majority of mitochondrial proteins are encoded in the nucleus, less is known about the opposite pathway, the so-called mitochondria-to-nucleus retrograde signaling pathway. Several studies have demonstrated that non-coding RNAs are essential “messengers” of this communication between the nucleus and the mitochondria and that they might have a central role in the coordination of important mitochondrial biological processes. In particular, the finding of numerous miRNAs in mitochondria, also known as mitomiRs, enabled insights into their role in mitochondrial gene transcription. MitomiRs could act as important mediators of this complex crosstalk between the nucleus and the mitochondria. Mitochondrial homeostasis is critical for the physiological processes of the cell. Disruption at any stage in their metabolism, dynamics and bioenergetics could lead to the production of considerable amounts of reactive oxygen species and increased mitochondrial permeability, which are among the hallmarks of cellular senescence. Extensive changes in mitomiR expression and distribution have been demonstrated in senescent cells, those could possibly lead to an alteration in mitochondrial homeostasis. Here, we discuss the emerging putative roles of mitomiRs in the bidirectional communication pathways between mitochondria and the nucleus, with a focus on the senescence-associated mitomiRs.

## Introduction

The aging process is considered a universal and inevitable process of physiological decline associated with a greater vulnerability to disease and death. This vulnerability is linked to the complexity of the organism, which comes from the myriad of interactions and feedback controls that operate between its different structural units. These mechanisms allow cells, tissues, and entire organisms the ability to respond and adapt to stressful environmental conditions. However, several studies suggest that this complexity diminishes with age due to the progressive loss of functions of cells, tissues, and organs and importantly of their ability to communicate, determining an increase of structural disorder; therefore, the complexity decrease is closely related to the increase in entropy, both determining the reduction of the functional reserve of older people (López-Otín et al., 2013). In this context, the Lorenz’s Butterfly metaphor makes the concept of “instability of the aging system” easier: even the slightest change can cause consequences that are not proportionate to the initial event; a small accident can induce fatal effects in the elderly or biologically old individual just as “a flapping of the wings of a butterfly in Brazil can trigger a hurricane in Texas” (Lorenz, 1972). During organismal aging, senescent cells accumulate in tissues, where they alter microenvironment homeostasis. Many theories of the origin of cellular senescence have started from the observation of microscopic changes in aging cells. López-Otín et al. (2013) tried to identify and categorize common cellular and molecular hallmarks of aging: stem cell exhaustion, genomic instability, telomere attrition, epigenetic alterations, loss of proteostasis, deregulated nutrient sensing pathways and mitochondrial dysfunction. Senescent cells exhibit several functional, phenotypic, and molecular changes including a stable arrest of proliferation. The metabolic alterations and changes in gene expression allow these cells to remain viable and resist to apoptosis for a long time but are also the cause of the acquisition of a common secretory phenotype. Cell cycle arrest in senescence is largely mediated *via* the activation of either one or both p16/pRB and p53/p21 tumor suppressor pathways. Prolonged overexpression of these four components is sufficient to induce senescence: pRB and p53 are key transcriptional regulators whereas p16 and p21 are cyclin-dependent kinase inhibitors which negatively regulate cell cycle progression. p53 and its downstream effector p21, are activated by DNA damage caused by oncogenic or oxidative stress and telomere attrition; however, epigenetically induced senescence mostly acts by inducing p16 expression that prevents phosphorylation of RB and thus the transcription of genes required for cell cycle progression (Kumari and Jat, 2021).

The secretory phenotype, called Senescence Associated Secretory Phenotype (SASP), and the altered intercellular communication are both important aspects of aging cells because they cause a low grade systemic, chronic inflammation called inflammaging (Franceschi et al., 2000). This condition plays a key role in the pathophysiology of inflammatory age-related diseases (ARDs), i.e., cancer, diabetes, cardiovascular and neurodegenerative diseases. Judith Campisi and her group first coined the term SASP and demonstrated that genotoxic stress-induced senescent cells secrete a myriad of factors associated with inflammation and oncogenesis (Coppé et al., 2008). Since then, scientific data on the characterization and the pathogenetic role of the SASP has increased enormously, but from our understanding, SASP is represented by the release of different soluble factors, regardless of the type of senescence, i.e., induced or replicative, pro-inflammatory cytokines, chemokines, and non-coding RNAs (ncRNAs), including small (microRNAs), long (lncRNA) and circular RNA (Terlecki-Zaniewicz et al., 2018; Mensà et al., 2020). The SASP can propagate signals (proteins, lipoproteins, DNA and RNA) at systemic levels, which contributes to the communication between different types of cells and tissues (Fafián-Labora and O'Loghlen, 2020). Consolidated data have revealed that NF-κB signaling is the major signaling pathway which stimulates the appearance of the SASP and the production of pro-inflammatory mediators (Salminen et al., 2012).

MicroRNAs (miRNAs or miRs) are small non-coding RNAs (sncRNAs), about 18–25 nucleotides long, which can modulate various physiological and pathophysiological processes at a post-transcriptional level by binding the 3'-untranslated region of the target mRNA in the cytoplasm, inhibiting its expression. Their biogenesis, which has been elegantly described by other authors (Bartel, 2004; Ha and Kim, 2014; Wang et al., 2017a; Treiber et al., 2019) occurs in multiple steps, both in the nucleus and the cytoplasm. After pri-miRNAs are transcribed, they are subsequently cleaved to the more stable form pre-miRNAs by Drosha. Then they translocate in the cytoplasm where they associate with Ago2, after Dicer processing. It is only at this stage that the RNA-inducing silencing complex (RISC) takes shape, thus the binding with the target mRNA. MicroRNAs are potentially involved in all cellular functions, including development, proliferation, differentiation, apoptosis, and aging. A multitude of genome wide expression profile experiments have shown a differential modulation of non-coding RNA, including miRNAs, between proliferating and senescent cells (Faraonio et al., 2012; Giuliani et al., 2020). Most of ncRNAs and miRNAs play a pivotal role in inducing cellular senescence and related organismal dysfunction. MiRNAs can be actively released by living cells, shuttled by proteins and/or extracellular vesicles (EVs) and internalized by target cells, which spreads specific signals at paracrine and systemic levels. Senescent cells *via* the release of EVs containing a number of senescence-associated (SA)-miRNAs can spread the senescent phenotype (Mariani et al., 2020; Mensà et al., 2020; Olivieri et al., 2021; Prattichizzo et al., 2021).

Several SA-miRNAs were shown to affect mitochondrial dynamics and bioenergetics. All known SA-miRNAs are coded by the nuclear genome and some of these have been found overexpressed within mitochondria of replicative senescent cells compared to younger, impacting on resident proteins and functions. The implications of nuclear-coded miRNAs targeting mitochondrial functions on the acquisition of the senescent phenotype (anterograde signals) and the onset of ARDs will be explored in this review. Furthermore, recent studies regarding retrograde signals (RTG) from mitochondria to the nucleus in the senescent cells will be discussed. The study of these relationships could be useful to figure out effective interventions in slowing aging and preventing ARDs.

## Mitochondria and Senescence

Mitochondria are involved in many processes besides energy metabolism such as cell cycle regulation, apoptosis and inflammation (Nunnari and Suomalainen, 2012). This suggests a close relationship between the proper performance of these organelles and cellular senescence. Mitochondrial dysfunction is in fact associated with the aging process and with the pathogenesis of ARDs. Some features of aged mitochondria, such as accumulation of mutation in mitochondrial DNA (mtDNA), altered mitochondria dynamics and increased reactive oxygen species (ROS) generation accompanied by progressive decrease in energy production, will be discussed below.

The mtDNA is circular and extremely small (16,569 nucleotides, 37 genes): it contains 13 mRNAs coding for some of the protein subunits of the oxidative phosphorylation (OXPHOS) machinery, two ribosomal RNAs (12S and 16S rRNAs) and 22 transfer RNAs (tRNAs; Carelli and Chan, 2014). However, about 3,000 genes are needed to make a mitochondrion and therefore their resulting proteins must be transported from the nucleus to the developing organelle. Only about 3% of the genes needed to make a mitochondrion are for ATP production. The remaining genes are involved in other functions related to the specialised tasks of the differentiated cells in which they reside. The integrity of mtDNA plays a key role in maintaining cellular homeostasis. Several studies carried out both on *in vitro* and *ex vivo* cellular models have demonstrated a close positive correlation between mtDNA mutations and the activation of mechanisms that lead to cellular dysfunction and, more generally, to the aging process (Trifunovic, 2006). We understand that mtDNA undergoes a higher mutation rate compared to nuclear DNA. In a neuronal stem cells model, for example, the accumulation of mtDNA damage led to a greater predisposition to differentiation towards an astrocytic lineage to a detrimental neurogenesis (Wang et al., 2011).

Mitochondrial dynamics, which vary through the life cycle of the cell depending on energy demands and cell division state, are critical to maintain mitochondrial integrity. During their life, which is about 10days, mitochondria are faced with fission and fusion (Hales, 2010). The former is required to remove depolarized, damaged, and dysfunctional mitochondria *via* autophagy (mitophagy). The second mechanism allows viable mitochondria maintenance since repolarized organelles can be recovered and restored by fusion with healthy elements of the mitochondrial network. During fusion events some functional components can be irregularly redistributed between mitochondria; as a consequence, dissimilar mitochondria can be generated by the next fission event (Westermann, 2010).

Several mitochondrial alterations, i.e., mtDNA mutation, ROS overproduction, depolarization, and misfolded protein lead to the accumulation of PTEN induced kinase 1 (PINK1) on outer mitochondrial membrane, which phosphorylates Parkin (PARK2). Parkin 2 in turn promotes the ubiquitination of proteins that are then recognised by the autophagic machinery. The autophagosome containing the dysfunctional mitochondria is then transported and fused to a lysosome where it is then degraded (Kazlauskaite and Muqit, 2015). However, during aging dysfunctional mitochondria accumulate, mainly for two reasons: autophagy declines (Wang and Klionsky, 2011), and fission overcomes fusion (Amartuvshin et al., 2020; Spurlock et al., 2020). Indeed, in senescent cells, the transcription factor p53 interacts with Parkin by inhibiting its accumulation on the outer membrane and consequently blocking the process of mitophagy (Hoshino et al., 2013; Badr et al., 2014; Correia-Melo et al., 2017; Manzella et al., 2018). Therefore, the number of mitochondria is greater in senescent cells than in young cells, but their functionality is severely compromised (Kim et al., 2018; Chapman et al., 2019).

We thoroughly explored the phenotype of mitochondria in human endothelial cells undergoing replicative senescence and observed elongated/branched morphology concomitantly with autophagic vacuole accumulation (Giuliani et al., 2018). Accordingly, Mai and co-workers suggest an hyper-fused state of the mitochondria due to downregulation of the fission regulating proteins, i.e., fission1 and dynamin-related protein 1 (Drp1; Mai et al., 2010). Similar mitochondrial morphology was observed in primary dermal fibroblasts induced to senescence with doxorubicin or hydrogen peroxide. These data show that mitochondrial hyperfusion can be associated with aging; this may appear contradictory with respect to the concept that mitochondrial fusion is an adaptive and protective response during stress. In this respect it has been suggested that in certain cell types an apparently compensatory mitochondria hyperfusion may have long-term negative consequences and accelerate aging.

This severe impairment of mitochondria function in senescent cells is also characterized by a higher basal oxygen consumption rate, which leads both to an increase in energy production and to a greater release of ROS (Hutter et al., 2004). Excessive ROS production provokes telomere shortening which culminates in the DNA-damage response, thus speeding up the senescence process (Passos et al., 2007; Rai et al., 2009; Hewitt et al., 2012).

Therefore, it has been postulated that mitochondrial morphology transitions might regulate mitochondrial function by RTG signaling (Picard et al., 2013; Walczak et al., 2017).

## Mitonuclear Communication

Mitochondria biogenesis is controlled by two physically separated genomes: the mtDNA and the nuclear genome. Therefore, an intense communication with the nucleus is required in order to provide mitochondria with nuclear-encoded proteins, which are necessary for mitochondrial homeostasis and function.

The coordination between the nucleus and the mitochondrion is mediated by a sophisticated communication system, named mitonuclear communication, which occurs in a bi-directional way, known as anterograde signaling (nucleus-to-mitochondrion) and retrograde signaling (mitochondrion-to-nucleus). The former mechanism reflects the accepted outlook of the nucleus as a regulatory factor, which coordinates the function of subcellular organelles, allowing mitochondria to adapt to the cellular milieu in response to endogenous alterations or extracellular stimuli. The retrograde (RTG) signaling is meant to be a feedback system of the mitochondrial functional state to the nucleus to initiate adaptive responses (Quirós et al., 2016; Bhatti et al., 2017). It might be considered as a quality control mechanism, which compensates for the loss of mitochondrial quality that naturally occurs with age.

### Anterograde Signaling

The anterograde signals are induced by extracellular stimuli, like physical exercise, cold exposure and dietary restrictions, through the activation of several genes including transcription factors, such as nuclear-respiratory factor 1 (NRF-1) and GA-binding protein-α (known as “NRF2”), peroxisome proliferator-activated receptors (PPARs); mitochondrial transcription factor A, uncoupling proteins, oestrogen-related receptors and PPARγ co-activator 1α (PGC1α; Hock and Kralli, 2009; Whelan and Zuckerbraun, 2013; Quirós et al., 2016). For example, during exercise or caloric restriction, there is an overall increase of AMP/ATP ratio, which in turn triggers AMP-activated protein kinase, along with an increment of NAD^+^ levels. This event leads to the activation of sirtuin 1 (SIRT1), a positive regulator of PGC1α, which is known to stimulate mitochondria metabolism and proliferation.

In the last few decades, there is growing evidence to underpin the involvement of ncRNAs in the regulation of mitochondrial homeostasis. Several research groups have examined their possible localization within mitochondria. In 2006, from an RNA sequencing experiment on rat’s liver mitochondria, a few miRNAs were identified inside the organelle, the so-called mitomiRs. Although initially thought as cytosolic contamination, just a few years later, several independent studies involving microarray profiling confirmed the initial findings (Lung et al., 2006; Kren et al., 2009; Bandiera et al., 2013).

**Figure 1:**
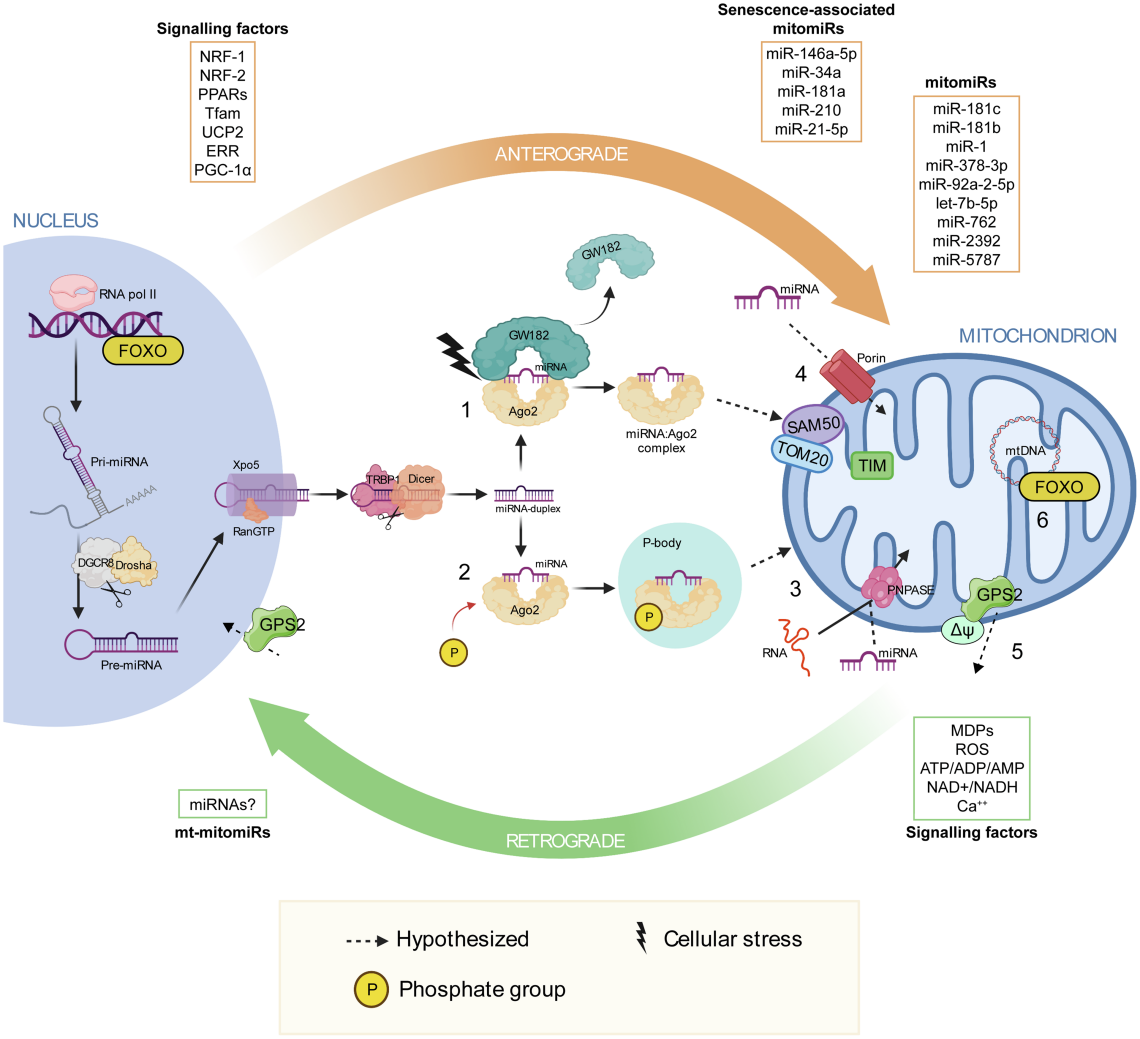
Mitonuclear communication with a focus on the proposed mechanisms of miRNA import into mitochondria. Mitonuclear communication plays a key role in mitochondrial homeostasis. The nucleus coordinates a number of mitochondrial functions through the so-called anterograde signaling, which involve various signaling factors, including nuclear-respiratory factor 1, nuclear-respiratory factor 2, peroxisome proliferator-activated receptors, mitochondrial transcription factor A, uncoupling proteins, ERR and PPARγ co-activator 1α. MicroRNAs have been observed in this communication network, some are transported into the mitochondria (mitomiRs) and a few of which are associated with cellular senescence associated-mitomiRs. At the same time, mitochondria communicate their functional and oxidative state to the nucleus *via* the retrograde response, which involve mitochondria-derived peptides and metabolites, such as ROS, ATP/ADP/AMP, NAD^+^/NADH and Ca^2+^. After their biogenesis in the nucleus and their translocation in the cytosol, miRNA duplexes are unwound, they bind to the Argonaute protein 2 (Ago2) and are loaded into the RNA-inducing silencing complex. (1) Due to cellular stress GW182 dislocates from RISC, leaving the miRNA:Ago2 complex free in the cytosol. It has been proposed that miRNA:Ago2 might be imported into mitochondria through the SAM50 and TOM20 pores in the outer membrane and TIM in the inner mitochondrial membrane (Jusic and Devaux, 2020). (2) Phosphorylation of Ago2 induces miRNA:Ago2 intake into a P-body, which is known to interact with mitochondria. (3) Translocation of miRNA:Ago2 complex into mitochondria may be supported by polynucleotide phosphorylase. Polynucleotide phosphorylase facilitates mRNA import into mitochondria, by binding to stem loop motifs in the RNA sequence. (4) Porins participate in miRNA translocation into mitochondria, as proposed by Bandiera et al. (2013). (5) After mitochondrial depolarization, GSP2 translocates into the nucleus, triggering the expression of genes involved in mitochondrial homeostasis. (6) Upon sensing stress stimuli in the mitochondrion, Forkhead box O can induce specific sets of nuclear genes, including autophagy effectors and antioxidant activators. Parts of the figure were realized using BioRender (http://biorender.com).

Extensive miRNA mapping analysis revealed that most of them are nuclear encoded, strengthening the assumption of a nuclear involvement in mitochondria homeostasis, which ultimately implies a miRNA import mechanism inside mitochondria. Only recently, we have started to discover mechanisms of RNA export and import into mitochondria, but none of which are miRNAs’ specific (Figure 1; Zeng et al., 2008; Wang et al., 2010; Jannot et al., 2016).

Since Ago2, an essential protein for RISC functioning, and some of its targets, such as some tRNA genes, have been localized into mitochondria (Bandiera et al., 2011), Bandiera and colleagues proposed an Ago2 involvement in the transport of miRNAs into mitochondria, as a protein import system similar to those involved in RNA trafficking. It has been demonstrated that phosphorylation of Ago2 induces miRNA:Ago2 complex intake into cytoplasmic processing bodies (P-bodies), which are known to interact with mitochondria (Zeng et al., 2008; Huang et al., 2011).

An important role is also played by GW-bodies in the formation of structures where the protein GW182 holds the association miRNA:Ago2 in a cap-like structure to make a stable RISC. A subsequent deletion of the cap and of the poly(A) tail, or the detachment of GW182 alone, presumably induced by cellular stresses, leaves miRNA:Ago2 complex free to head towards mitochondria (Gibbings et al., 2009). The proposed mechanism of miRNA:Ago2 entry involves gates such as SAM50, TOM20 and TIM (Jusic and Devaux, 2020).

Another import mechanism might involve the polynucleotide phosphorylase PNPASE, a 3'→5' exoribonuclease and poly-A polymerase located in the mitochondrial intermembrane space, that regulates mitochondrial homeostasis and adenine nucleotide levels (Wang et al., 2010). Wang and co-worker showed how PNPASE imports RNA from the cytosol into the mitochondrial matrix by binding to specific stem-loop motifs in the RNA sequence, making assumption on its potential involvement in mitochondrial miRNA trafficking (Wang et al., 2012).

Finally, porins, highly conserved proteins located in the outer membrane of mitochondria, could participate in miRNA translocation from cytosol to mitochondria, as proposed by Bandiera et al. (2013).

Notably, there is increasing body of evidence to suggest that mitochondria or mtDNA are exposed to intracellular or intercellular transfer *via* exosome and tunnelling nanotubes (Spees et al., 2006; Borralho et al., 2014).

It is widely accepted that housekeeping mitochondrial nuclear-encoded ncRNAs regulate mitochondrial function and homeostasis (Gusic and Prokisch, 2020), but less is known about their deregulation and possible correlation with mitochondrial-related diseases. Several nuclear-encoded lncRNAs have been localized in mitochondria and it has been hypothesized that they could regulate mitochondrial functions (Vendramin et al., 2017). It has been demonstrated that SAMMSON, a nuclear-encoded lncRNA acts as an oncogene in several malignancies (Leucci et al., 2016; Zheng et al., 2020), as well as MALAT1, which is known to be associated with cancer and metastasis (Sun and Ma, 2019). However, it is still an uncovered field, since not much data are available and mitochondria import mechanisms have still to be elucidated; therefore, the presence and the function of these transcripts in mitochondria, especially during cellular senescence, need further investigation. As mentioned above, different nuclear-encoded ncRNAs, such as miRNAs, are involved in mitochondrial homeostasis and are deregulated during senescence. The section “Anterograde signaling *via* nuclear-encoded mitomiRs in ARDs” will deeply describe nuclear-encoded miRNAs and their impact in mitochondria function and dysregulation.

### Retrograde Signaling

The retrograde signaling pathway is activated when dysfunctional mitochondria try to communicate their oxidative, metabolic and respiratory stressful conditions to the nuclear compartment, thus inducing a wide range of cellular adaptations (Passos et al., 2007; Quirós et al., 2016).

The most well studied retrograde signals are pleiotropic and include ROS, pro-apoptotic molecules such as ATP/ADP/AMP and metabolites such as NAD^+^/NADH and Ca^2+^ (Picard et al., 2013), which, however, lack specificity as signaling molecules. Thus, the exact mechanism underlying the retrograde signaling by which mitochondria regulate cellular processes has not been completely unravelled yet.

Recently, mitochondrial-derived peptides (MDPs) encoded by a short open reading frame into mtDNA have been identified. Three types of MDPs have been discovered so far, including Humanin, MOTS-c and SHLP1-6. Their role is associated with cell survival, metabolism, inflammation and response to stressors with the final aim of maintaining mitochondrial function under stress conditions (Yang et al., 2019; Conte et al., 2021).

Recent reports highlight that the mammalian mitochondrial genome, in addition to the 37 known mitochondrial genes, encodes several classes of sncRNAs, “mitochondrial genome-encoded small RNAs” (mitosRNA), whose deregulation have been correlated with dysfunctional mitochondria and ultimately with a variety of diseases (Ro et al., 2013). To examine mitosRNA biogenesis, the presence of a mitochondrial RNAi machinery was investigated by Ro and colleagues, but neither Dicer nor AGO2 expression was detected. Therefore, mitosRNAs do not derive from RNA turnover, but they must be products of unknown mitochondrial ribonucleases (Ro et al., 2013).

NGS analyses performed by Larriba et al. (2018), who classified mitosRNAs into several groups of sncRNAs, emphasized the predominance of Piwi-interacting RNAs compared to other sncRNA categories, with regulatory functions in mitochondria and important for gametes and zygotic cells development, showing cell-type specific expression.

Despite mitosRNAs exact biogenesis and cellular trafficking are still uncertain (Vendramin et al., 2017), many studies about their possible function were carried out. Of note, mito-ncR-805 has been shown to have a protective effect to cigarette smoke in alveolar epithelial cells, as well as an increased expression of nuclear-encoded genes important for mitochondrial function, supporting the idea that mitosRNAs regulation and function might be cell-type specific (Blumental-Perry et al., 2020).

How mitosRNAs are exported from mitochondria and imported into the nucleus is still not known (Gammage et al., 2018), however studies about the expression of sense mitochondrial ncRNAs (SncmtRNAs) and antisense mitochondrial ncRNAs (ASncmtRNAs) between normal and cancer cells, demonstrated a cytoplasmic and nuclear localization of these mitochondrial transcripts, reinforcing the concept that they are exported from mitochondria (Villegas et al., 2007; Landerer et al., 2011; Borgna et al., 2020).

A direct mitonuclear communication strategy for mammals, similar to those found in yeasts and worms (Jazwinski and Kriete, 2012; Nargund et al., 2012, 2015), has been proposed by Cardamone et al., who characterized the G-Protein Pathway Suppressor 2 (GPS2; Cardamone et al., 2017), which also regulates insulin signaling, lipid metabolism and inflammation (Jakobsson et al., 2009). Cardamone and colleagues demonstrated that upon mitochondrial depolarization, GPS2 translocates into the nucleus, triggering the expression of genes involved in mitochondrial homeostasis. Other transcription factors involved in retrograde signaling have been identified, such as Forkhead box O (FOXO), which is known to help in the transcription of mitochondrial antioxidant enzymes, inducing mitophagy (Kim and Koh, 2017).

However, to our knowledge, since the details of this complex pathway are still unexplored, not much is known about other RNA export mechanisms from mitochondria, and if these also involve mitosRNAs.

New data support the idea of a mitonuclear communication involvement in aging. Since Ca^2+^ is the most important signaling molecule in the retrograde signaling pathway and studies in senescent human MRC5 fibroblasts correlated with increased mitochondrial biogenesis and Ca^2+^ alterations, it has been proposed that dysfunctional mitochondria might communicate with the nucleus *via* calcium signaling (Passos et al., 2007). Moreover, several studies reported that during induced or replicative senescence, mitochondria produce a higher amount of MDPs (humanin and MOTS-c), which can regulate mitochondrial energy metabolism, playing a cytoprotective role in ARDs. For example, humanin has a crucial role in reducing oxidative stress, while MOTS-c in glycolipids metabolism protects endothelial cells from atherosclerosis (Kim et al., 2018). Additional researchers have outlined that the increased production of ROS by defective mitochondria induces cytoplasmic chromatin fragments formation, which is JNK kinase-mediated, which is a trigger of SASP (Vizioli et al., 2020).

As far as we know, only a few studies have outlined the possibility of a relationship between mitosRNAs and ARDs. For example, SncmtRNA-1, SncmtRNA-2, ASncmtRNA-1 and ASncmtRNA-2 have been associated with cancer (Burzio et al., 2009; Vidaurre et al., 2014; Villota et al., 2019), whereas mt-lncRNA has been correlated with cardiovascular diseases (Yang et al., 2014). An interesting work by Shinde and colleagues (Shinde and Bhadra, 2015), demonstrates the expression of six novel mitochondrial genome-encoded miRNAs (mt-mitomiRs; Giuliani et al., 2019) in mitochondria and that the MT-RNR2 gene could be a potential target of two of them, hsa-miR-mit-3 and hsa-miR-mit-4. Curiously, MT-RNR2 gene also encodes for the humanin peptide, which has been correlated with Alzheimer’s disease (Tajima et al., 2002), indicating a potential involvement of them in the development of the disease. MitosRNA association with ARDs makes them promising biomarkers of the diseases, therefore future studies will be required for a potential application in clinical practice.

## Anterograde Signaling *Via* Nuclear-Encoded mitomirs In Ards

One of the first piece of evidence that nuclear-encoded miRNA can regulate the expression of mitochondrial genome was provided by Das and colleagues. The authors showed that after its maturation in the cytoplasm, miR-181c translocated in the mitochondrial compartment, where its principal target is the mt-COX-1 gene. Since mitochondrial DNA is sequentially transcribed as a polycistronic unit, miR-181c affects multiple proteins, including mt-COX2 and mt-COX3, resulting in complex IV remodelling (Das et al., 2012). Being the last complex of the respiratory chain, complex IV plays an important role in the transfer of electrons from cytochrome c oxidase to oxygen. Mutations or deregulation of complex IV lead to higher levels of ROS and mitochondrial dysfunction and are associated with a negative impact on lifespan and tissue integrity (Reichart et al., 2019). Cardiac myocytes are the cells with the highest volume density of mitochondria in the body and rely their extraordinary demand for continuous energy production on oxidative metabolism. As a result, these cells have been used to unravel mitomiR role in mitochondrial homeostasis. Das and co-workers confirmed their results *in vivo* using a lipid-based cationic nanoparticles miR-181c delivery system, which demonstrated that chronic overexpression of miR-181c is involved in heart failure (Das et al., 2014). Besides miR-181c, other miR-181 family members – i.e. miR-181a -181b, -181d – are found in mitochondria and have implications in heart mitochondrial health (Das et al., 2017). Interestingly, miR-181a and miR-181b have been shown to exert divergent roles in myocardial function. At the early stages of heart failure, miR-181a and -181b are consistently upregulated in cardiomyocyte mitochondria whereas, at the later stages, only miR-181b levels tend to remain stable, in association with a downregulation of miR-181a (Wang et al., 2017b). In this framework, mitomiRs acquire a critical role in cardiac function. Another miRNA widely studied in cardiac and skeletal muscle tissues is miR-1, which is actively involved in myogenesis and muscle proliferation (Chen et al., 2006). Increased expression of miR-1 was found in aging hearts, suggesting that this miRNA participates in additional cellular or pathophysiological functions other than myogenesis (Yang et al., 2007). MiR-1 has several cytosolic targets; however, during muscle differentiation it translocates to the mitochondria where it, surprisingly, enhances translation of mt-COX-1 and mt-ND1, resulting in boosted ATP generation. Zhang and colleagues showed that overexpression of miR-1 could have a negative impact on mitochondrial morphology and physiology in cancer stem cells, by targeting nuclear-encoded proteins required for mitochondria organization (Zhang et al., 2019). The non-canonical role as a translational activator of miR-1 seems to be linked to the lack of miRNA-mediated gene silencing GW182 inside the mitochondria, suggesting that the miRNA machinery is rearranged in this organelle (Zhang et al., 2014).

MitomiRs are also emerging players in the pathogenetic processes of diabetic heart diseases. Diabetes is associated with cardiac functional deficits, which may result from a decreased mitochondrial ATP output. Jagannathan et al. analyzed mitomiR distribution in the two spatially distinct mitochondrial subpopulations, i.e., subsarcolemmal and interfibrillar mitochondria, following diabetic insult. Of particular interest is mitomiR-378-3p, which originates from the first intron of peroxisome proliferator-activated receptor gamma, coactivator 1 beta gene that encodes PGC1β. This miRNA has been implicated in lipid metabolism, mitochondrial function, and shift towards the glycolytic pathway (Carrer et al., 2012; Krist et al., 2015). MitomiR-378 binds the ATP synthase F0 subunit 6 (ATP6), leading to a drop of ATP production following diabetes insult in interfibrillar mitochondria of mice (Jagannathan et al., 2015). In a condition of diabetic cardiomyopathy, mitomiR-92a-2-5p and let-7b-5p enter into the mitochondria to counteract cytochrome-b downregulation. Overexpression of miR-92a-2-5p enhances mitochondrial translation and reduces ROS production and lipid deposition, rescuing cardiac diastolic dysfunction in the db/db mouse model (Li et al., 2019). Also, miR-762 translocates to mitochondria upon ischemia/reperfusion model and downregulates ND2 leading to inhibition of ATP production and the enzyme activity of complex I, induction of ROS generation and apoptotic cell death in cardiomyocytes (Yan et al., 2019).

Metabolic reprogramming is a feature of cancer cells. Two interesting papers showed how mitomiRs can promote chemotherapy resistance by inducing glycolysis in cancer cells. Nuclear-encoded mitomiR-2,392 and -5,787 are involved in reprogramming metabolism *via* increase of glycolysis and inhibition of OXPHOS, resulting in enhanced chemoresistance in tongue squamous cell carcinoma cells (Chen et al., 2019; Fan et al., 2019).

The mitomiR-mediated switch of energy sources during cellular differentiation suggests a pivotal role of mitomiRs in all processes requiring metabolic reprogramming, including cellular senescence (Sabbatinelli et al., 2019). Indeed, during senescence mitochondrial function declines, which creates an energy deficit. Retrograde signaling tries to overcome this energy deficit by increasing mitochondrial biogenesis as well as glycolysis, as a compensatory measure. However, this compensation is partial and accompanied by an increase in ROS production, thus creating a cycle of further damage to the mitochondria itself and to the cell.

Overall, we can conclude that mitomiRs can interact with mitochondrial genome in multiple ways: (i) as prompt compensators after a negative insult — e.g., miR-92a-2-5p and let-7b-5p, (ii) as effectors of mitochondrial dysfunction — e.g., miR-738-3p, (iii) as mediators of physiological cellular processes — e.g., miR-1.

## Senescence-Associated Mirnas Impact on Mitochondrial Function

Senescence associated-mitomiRs have been demonstrated to affect all aspects of mitochondrial homeostasis. While the role of SA-mitomiRs on the expression of mitochondrial genes has yet to be elucidated, multiple pieces of evidence support their ability in modulating several processes linked to mitochondrial function.

Through an *in silico* analysis we have suggested that SA-mitomiRs may affect endothelial cell sensitivity to apoptosis through Bcl2 family member regulation (Rippo et al., 2014). Furthermore, we have recently demonstrated that miR-146a-5p, miR-34a and miR-181a levels are increased during replicative senescence of endothelial cells and enriched in senescent mitochondria. Their overexpression induces permeability transition pore opening, ROS production, caspase-1 and -3 activation and autophagic vacuole accumulation at least due to Bcl-2 downregulation (Giuliani et al., 2018).

MiR-146a is one of the most extensively studied miRNAs in the field of senescence and inflammation (Olivieri et al., 2013a,b) The synthesis of miR-146a is intimately linked to inflammatory processes and its effects on cellular processes are highly stimulus- and context-dependent. Indeed, miR-146a, which is particularly enriched in the mitochondrial fraction of cardiomyocytes, exerts a cardioprotective role by inhibiting the mitochondria-dependent apoptotic pathway and attenuating the loss of mitochondrial membrane potential. Evidence from cardiomyocyte-specific knockout and overexpression experiments supported the hypothesis that adequate miR-146a levels are required to reduce the extent of myocardial infarction and cardiac dysfunction following ischemia/reperfusion damage (Su et al., 2021). Notably, acute cellular damage can affect the trafficking of miRNAs between the mitochondrial and cytosolic compartments. Following a severe traumatic brain injury (TBI), miR-146a-5p levels decrease in the hippocampal mitochondrial fraction, in association with an increase of its cytosolic expression. This compartmental shift was shown to be triggered by decreased mitochondrial bioenergetics following TBI.

MiR-146a cytosolic enrichment avoids uncontrolled activation of the NF-kB pathway by targeting its upstream members TRAF6 and IRAK1. Mitochondria can act as first-line responders to cellular stressors by triggering pathways leading to altered nuclear gene expression, also by affecting miRNA intracellular localization (Wang et al., 2015, 2021). Interestingly, miR-146a-5p also impacts mitochondrial dynamics by targeting PARK2, one of proteins involved in mitophagy. Decreased amounts of PARK2 lead to the accumulation of damaged and dysfunctional mitochondria, which exacerbates the ROS-induced neuronal damage (Jauhari et al., 2020). Parkin 2 is a component of a multiprotein E3 ubiquitin ligase complex that mediates the targeting of substrates, including mitochondrial proteins, for proteasomal degradation and mitophagy. Interestingly, PARK2 mutations have been associated with early onset Parkinson’s disease (Kitada et al., 1998). Besides its neuroprotective and cardioprotective function, a role in liver homeostasis has been demonstrated for miR-146a. Its hepatocyte expression is needed to promote the mitochondrial oxidation of fatty acids and improve insulin sensitivity, which prevents the detrimental lipid accumulation in the liver. These effects are achieved by promoting both mitochondrial biogenesis and synthesis of electron transport chain complex subunits through targeting of MED1, a component of the mammalian mediator complex involved in adipogenesis and mitochondrial gene expression (Li et al., 2020). Moreover, bioinformatic analysis identified multiple miR-146a potential targets in mitochondrial genome, suggesting there is still much to uncover about the crosstalk between miR-146a and bioenergetic pathways (Dasgupta et al., 2015; Giuliani et al., 2017).

MiR-34a-5p has been implicated in the pathogenesis of ARDs accompanied by mitochondrial dysfunction and impairment of the autophagic flux, such as neurodegenerative disorders. MiR-34a-5p targets PINK1, a stress sensor that localizes to the outer mitochondrial membrane following the loss of mitochondrial potential. This triggers mitochondrial clearance. Similarly to PARK2, also PINK1 mutations have been involved in the pathogenesis of early onset Parkinson’s disease. In this framework, overexpression of miR-34a-5p attenuates mitochondrial protein ubiquitination and prevents the recruitment of PARK2, thus delaying mitophagy (Tai et al., 2021). One of the main targets of miR-34a with a well-established role in aging is SIRT1. The rescue of SIRT1 levels following miR-34a inhibition reduced age-related hearing loss in C57BL/6 mice. Although the full signaling pathway responsible for this effect has still to be elucidated, the miR-34a/SIRT1 axis was hypothesized to affect the balance between mitophagy and mitochondrial biogenesis and to protect cochlear cells against oxidative stress-mediated apoptosis (Xiong et al., 2019). MiR-34a was also shown to accelerate renal aging by affecting the mitochondrial function of mesangial cells. MiR-34a targets the mRNA of thioredoxin reductase 2, a protein involved in the scavenging and detoxification of mitochondrial ROS. The levels of miR-34a were particularly high in senescent mesangial cells and, at the same time, miR-34a overexpression induced premature senescence in these cells due to the accumulation of dysfunctional mitochondria (Bai et al., 2011).

The link between mitochondrial function and miR-181a has been elucidated in multiple cellular models (Mancini et al., 2012; Indrieri et al., 2020). Interestingly, miR-181a is among the most characterized miRNAs in lymphoid tissue, with a well-documented role in T cell aging and immune senescence (Ye et al., 2018; Kim et al., 2021). The age-related decline in miR-181a expression in naive and memory T cells may account for some of the age-associated defects in T cell function. To this regard, restoration of miR-181a intracellular levels provided a feasible strategy to boost T cell response in the elderly (Li et al., 2012; Ye et al., 2018, 2021). Recently, it also has been shown a decline of miR-181a-5p in NK cells from the aged mice, impairing the production of IFN-γ (Lu et al., 2021). Moreover, miR-181a shows an age-dependent decline in peripheral blood mononuclear cells from donors of different ages (Xu et al., 2020). Similarly to miR-34a, miR-181a is a regulator of mitochondrial dynamics through the action on several proteins implied in the mitophagy process. Restoration of the expression of miR-181a in the skeletal muscle of old mice improved mitochondrial quality (Goljanek-Whysall et al., 2020). The prominent role of miR-181a-5p in age muscle homeostasis was elegantly reviewed by Borja-Gonzalez and colleagues (Borja-Gonzalez et al., 2020). MiR-181a is closely associated to inflammation and apoptosis in neuronal cells, where it targets mitochondria-related proteins, i.e., heat shock protein 70, glucose regulated protein 78, anti-apoptotic Bcl-2, and myeloid cell leukemia-1 (Ouyang et al., 2012; Hutchison et al., 2013).

MiR-210 is considered the master hypoxia-related miR, because of its prompt upregulation under hypoxia in most cell types. MiR-210 is upregulated in senescent cells where it is involved in double-strand DNA breaks and ROS accumulation (Faraonio et al., 2012) *via* the inhibition of the electron transport chain (ETC) protein translation (Karshovska et al., 2019). MiR-210 directly targets NDUFA4 and SDHD — subunits of the ETC complex I and II, respectively — and induces mitochondrial dysfunction (Puisségur et al., 2011). Future studies are warranted to unravel its specific activity in mitochondrial function.

MiR-21, initially classified as an ‘onco-miR’ due to its modulation in different types of cancer, has an extensively established role in inflammatory and senescence processes (Olivieri et al., 2021). Increased expression of miR-21-5p was found in replicative and stress-induced models of senescence (Dellago et al., 2013; Mensà et al., 2020).

A particular myocardial enrichment of miR-21-5p has been observed in several models of cardiovascular disease, cardiac dysfunction, and heart failure, where it has been reported to prevent cardiomyocyte apoptosis by targeting the PDCD4 mRNA (Qin et al., 2012). *In vivo* silencing of miR-21, using a specific antagomir, has been found to attenuate cardiac fibrosis and cardiac dysfunction in pressure-overloaded hearts (Thum et al., 2008). Overexpression of miR-21 decreases mitochondrial fatty acid oxidation and concomitant mitochondrial respiration in rat cardiomyocytes, suggesting that miR-21 coordinates the shifting of cellular metabolism towards the glycolytic pathway (Nasci et al., 2019). This hypothesis was later confirmed by the evidence that miR-21 is able to translocate into the mitochondria and target mt-Cyb to enhance its translation in a spontaneous hypertensive rat model (Li et al., 2016). Moreover, miR-21-5p affects mitochondrial dynamics in a model of oxidized LDL-induced endothelial cell senescence by targeting Drp1 protein (Zhang et al., 2017).

Although miR-21 is also involved in several mitochondrial functions, the precise role of miR-21 in senescent mitochondria is far from being elucidated. Table 1 summarizes the mitomiR targets with a well-recognized role on senescence. The involved pathways are functional to the acquisition of the senescent phenotype, including cell cycle arrest, mitochondrial dysfunction, and production of pro-inflammatory cytokines, that is, SASP.

**Table 1:** Shows senescence-associated mitomiR targets.

SA-mitomiRs	Cytosolic target	Effect on senescence-related pathways	Mt-DNA target	Effect on mitochondrial functions
miR-21-5p	-NFIB and CDC25A (Dellago et al., 2013) -TLR8 (ligand; Zhang et al., 2018) -A20 (Xue et al., 2019) -PTEN (Buscaglia and Li, 2011; Ma et al., 2013) -PDCD4 (Matsuhashi et al., 2019)	-Cell proliferation arrest-Pro-inflammatory cytokine production-Activation of NF- κB pathway and NLRP3 inflammasome	-mt-Cyb (Li et al., 2016)	-enhanced mitochondrial translation
miR-146a-5p	-TLR4 (Xiao et al., 2019) -TRAF6 (Taganov et al., 2006) -IRAK1 (Taganov et al., 2006) -BCL2 (Giuliani et al., 2018)	-Pro-inflammatory cytokine production-Apoptosis sensitivity alteration-Activation of NF-κB pathway	-mt-ND1, mt-ND2, mt-ND4, mt-ND5, mt-ND6; -mt-ATP8 (PREDICTED; Dasgupta et al., 2015)	
miR-181a-5p	-SIRT1 (Di Val Cervo et al., 2012) -BCL2 (Giuliani et al., 2018) -PARK2 and p62/SQSTM1 (Goljanek-Whysall et al., 2020)	-Cell proliferation arrest-Apoptosis sensitivity alteration-Impaired autophagy		
miR-210	-NDUFA4 and SDHD (Puisségur et al., 2011) -ISCU1/2 (Chan et al., 2009)-RAD52 (Crosby et al., 2009) -E2F3 (Biswas et al., 2010)	-Mitochondrial dysfunction-DNA repair loss - Cell proliferation arrest		
miR-34a	-SIRT1/P53 axis-BCL2 (Giuliani et al., 2018) **-**Txnrd2 (Bai et al., 2011)	-Apoptosis sensitivity alteration-Cell proliferation arrest-Pro-inflammatory cytokine production-Increased oxidative stress		

*Tumor necrosis factor, alpha-induced protein 3(A20); B-cell lymphoma 2 (Bcl-2); Cell division cycle 25 A(CDC25A); E2F transcription factor 3 (E2F3); Interleukin 1 Receptor Associated Kinase 1 (IRAK1); Iron–sulfur cluster assembly enzyme (ISCU); mtDNA-encoded cytochrome b (mt-Cytb); Nuclear factor 1 B-type (NFIB); Phosphatase and tensin homolog (PTEN); succinate dehydrogenase complex subunit D (SDHD); Sirtuin 1 (SIRT1); Sequestosome-1 (SQSTM1); Toll like receptor (TLR); TNF Receptor Associated Factor 6 (TRAF6); Thioredoxin Reductase 2 (Txnrd2)*.

## Conclusion

The central role of mitochondria in cellular senescence is now a dogma recognized by all gerontologists in the world. Many biochemical and morphological changes to which these organelles meet are common to both stress-induced or replicative senescence: branching and elongation, ROS production, mtDNA mutations and membrane depolarization. Several of these phenomena are due to the sophisticated communication system (anterograde and retrograde signaling) between the nucleus and the mitochondrion. While an increasing number of research reports are shedding light on the anterograde signaling routes, retrograde signaling mechanisms are almost completely unknown although there is the certainty that they play important roles. Nuclear-encoded miRNAs shuttle within mitochondria, and at the epigenetic level, regulate both mt-DNA encoded proteins and those encoded by nuclear genes that are functional in mitochondria.

Since the mitochondrial alterations observed in the senescent cells represent etiopathogenetic factors in age-associated diseases, the deepening of the communication routes between nucleus and mitochondria may lead to devise new preventive and therapeutic strategies.

## Author Contributions

CG, AS, and AG performed literature search, drafted the manuscript and prepared the figure. MR conceived the idea and participated in manuscript drafting. FO reviewed the manuscript. All authors approved the final version of the manuscript.

## Funding

The present work has been supported by grants from Università Politecnica delle Marche, Italy to MR.

## Conflict of Interest

The authors declare that the research was conducted in the absence of any commercial or financial relationships that could be construed as a potential conflict of interest.

## Publisher’s Note

All claims expressed in this article are solely those of the authors and do not necessarily represent those of their affiliated organizations, or those of the publisher, the editors and the reviewers. Any product that may be evaluated in this article, or claim that may be made by its manufacturer, is not guaranteed or endorsed by the publisher.

## Abbreviations

ARDs: Age-related diseases
AMPK: AMP-activated protein kinase
DDR: DNA damage response
EVs: extracellular vesicles
Drp1: dynamin-related protein 1
ETC: electron transport chain
FOXO: forkhead box O
GPS2: G-Protein Pathway Suppressor 2
lncRNA: long non-coding RNA
miRNA, miR: microRNA
MDPs: mitochondrial-derived peptides
mtDNA: mitochondrial DNA
mitosRNA: mitochondrial genome-encoded small RNA
NF-κB: nuclear factor kappa-light-chain-enhancer of activated B cells
ncRNA: non-coding RNA
NRF-1: nuclear-respiratory factor 1
ORF: open reading frame
OXPHOS: oxidative phosphorylation
PARK2: Parkin
piRNA: Piwi-interacting RNA
PPAR: peroxisome proliferator-activated receptor
PGC1α: PPARγ co-activator 1α
PINK1: PTEN induced kinase 1
pRB: retinoblastoma protein
RTG: retrograde
RISC: RNA-inducing silencing complex
ROS: reactive oxygen species
SA: senescent-associated
SASP: Senescence Associated Secretory Phenotype
SncmtRNA: sense mitochondrial ncRNA
ASncmtRNA: antisense mitochondrial ncRNA
SIRT1: sirtuin 1
sncRNA: small non-coding RNA
Tfam: mitochondrial transcription factor A
p53: tumor protein P53
UTR: untranslated region
